# Emerging Role and Therapeutic Potential of lncRNAs in Colorectal Cancer

**DOI:** 10.3390/cancers12123843

**Published:** 2020-12-19

**Authors:** Laura Schwarzmueller, Oscar Bril, Louis Vermeulen, Nicolas Léveillé

**Affiliations:** 1 Laboratory for Experimental Oncology and Radiobiology, Center for Experimental and Molecular Medicine, Cancer Center Amsterdam and Amsterdam Gastroenterology and Metabolism, Amsterdam UMC, University of Amsterdam, Meibergdreef 9, 1105 AZ Amsterdam, The Netherlands; l.j.schwarzmuller@amsterdamumc.nl (L.S.); o.t.bril@amsterdamumc.nl (O.B.); l.vermeulen@amsterdamumc.nl (L.V.); 2 Oncode Institute, Meibergdreef 9, 1105 AZ Amsterdam, The Netherlands

**Keywords:** colorectal cancer, lncRNAs, RNA-based therapeutics

## Abstract

**Simple Summary:**

Homeostasis of the intestine is maintained by a delicate balance of signaling networks that regulate self-renewal and differentiation. In the past years, increasing evidence suggests that long non-coding RNAs (lncRNAs) are involved in the control of intestinal crypt turnover. Indeed, their deregulation can enable and drive malignant cell growth. Notably, lncRNAs have high tissue specificity, and therefore hold great potential for therapeutic intervention. Here, we address the function of lncRNAs in the intestine in physiological and pathological conditions and discuss promising interference systems to target oncogenic lncRNAs.

**Abstract:**

Maintenance of the intestinal epithelium is dependent on the control of stem cell (SC) proliferation and differentiation. The fine regulation of these cellular processes requires a complex dynamic interplay between several signaling pathways, including Wnt, Notch, Hippo, EGF, Ephrin, and BMP/TGF-β. During the initiation and progression of colorectal cancer (CRC), key events, such as oncogenic mutations, influence these signaling pathways, and tilt the homeostatic balance towards proliferation and dedifferentiation. Therapeutic strategies to specifically target these deregulated signaling pathways are of particular interest. However, systemic blocking or activation of these pathways poses major risks for normal stem cell function and tissue homeostasis. Interestingly, long non-coding RNAs (lncRNAs) have recently emerged as potent regulators of key cellular processes often deregulated in cancer. Because of their exceptional tissue and tumor specificity, these regulatory RNAs represent attractive targets for cancer therapy. Here, we discuss how lncRNAs participate in the maintenance of intestinal homeostasis and how they can contribute to the deregulation of each signaling pathway in CRC. Finally, we describe currently available molecular tools to develop lncRNA-targeted cancer therapies.

## 1. Introduction

Intestinal tissue homeostasis is regulated by a variety of signaling pathways influencing cell proliferation and differentiation. Through collaborative or antagonistic activity, these pathways adjust cellular states along the crypt–villus axis. Indeed, the intestine is organized into a succession of self-renewing protrusions and invaginations respectively known as villi and crypts [1] that increase surface area and improve its absorptive capacity. The intestine is lined with different types of epithelial cells, essential for both secretory and absorptive functions, as well as for the establishment of a physical and biochemical barrier that separates the gut lumen from the lamina propria. Intestinal stem cells (ISCs), located at the bottom of the crypts, drive the renewal of the epithelial cell layer as frequently as every 4–5 days [2]. The neighboring Paneth cells are key contributors in this process by providing soluble factors (e.g., epidermal growth factor (EGF), transforming growth factor alpha (TGFα), and Wnt family member 3 (WNT3)) essential for the ISC niche. However, maintaining tissue homeostasis is a complex task, and as such it relies on a variety of signaling pathways. For instance, Wnt and Notch promote the stem cell niche, while bone morphogenetic protein (BMP) and hedgehog (Hh) negatively regulate the expansion of stem cells, thus delineating the stem cell niche and favoring differentiation [3].

Three decades ago, Fearon and Vogelstein proposed the sequential accumulation of mutations as an essential oncogenic driving force during the initiation and progression of colorectal cancer [4]. Some of these mutations in key signaling pathways enable cells to become independent from stem cell niche factors [5]. For example, somatic mutations in the adenomatous polyposis coli (*APC*) gene, an essential factor for balancing Wnt activity, are found in approximately 80% of all colorectal carcinomas. Most of these mutations generate truncated versions of APC with reduced capacity to label β-catenin for degradation, resulting in the hyperactivation of Wnt signaling. Intracellular accumulation of β-catenin eventually leads to its nuclear translocation and consequent activation of the Wnt transcriptional program, thereby promoting proliferation and expansion of undifferentiated cells [6]. Alternatively, disruption of the BMP/TGF-β signaling pathway (e.g., mutations of *TGFBR2* and *SMAD4*) promotes tumorigenesis by desensitizing cancer cells from the growth inhibitory signals conveyed by the ligands [7,8,9].

Up to now, signaling pathways controlling maintenance and deregulation of intestinal tissue homeostasis have mostly been studied for their regulatory impact on protein coding genes. It is established that while only around 2% of the human genome is occupied by protein-coding genes, more than 70% of the human genome is transcribed [9]. This indicates the potential influence of non-coding RNAs (ncRNAs) in most if not all cellular functions. Apart from the well-studied small non-coding RNAs, long non-coding RNAs (lncRNAs) emerged as an important class of functional molecules. LncRNAs are RNA transcripts of more than 200 nucleotides with little to no protein-coding capability, and lower expression levels but higher tissue specificity than protein-coding transcripts [10,11,12,13]. Mechanistically, lncRNAs seem to exert their functions in the nucleus as well as in the cytoplasm, by interacting with nucleic acids, proteins, or lipids [14,15,16,17,18,19,20].

Intense efforts in the past decade have helped to demonstrate the crucial role of lncRNAs in the normal intestine and CRC. Indeed, many CRC-deregulated lncRNAs, including H19 [21], UCA1 [22], ROR [23], HOTAIR [24], PANDAR [25], and PVT1 [26], exert key functions that support tumor growth and progression (see the review [27]). In addition, their propensity to display tissue- or even cancer-specific expression profiles could be exploited for the development of novel RNA-based therapies. In this review, we will examine the role of lncRNAs across signaling pathways known to influence intestinal tissue homeostasis. We will further reveal how lncRNAs can contribute to signaling deregulation in CRC and explore their potential as therapeutic targets.

## 2. Signaling in the Intestinal Crypt and lncRNAs

### 2.1. Wnt Signaling

**Figure 1:**
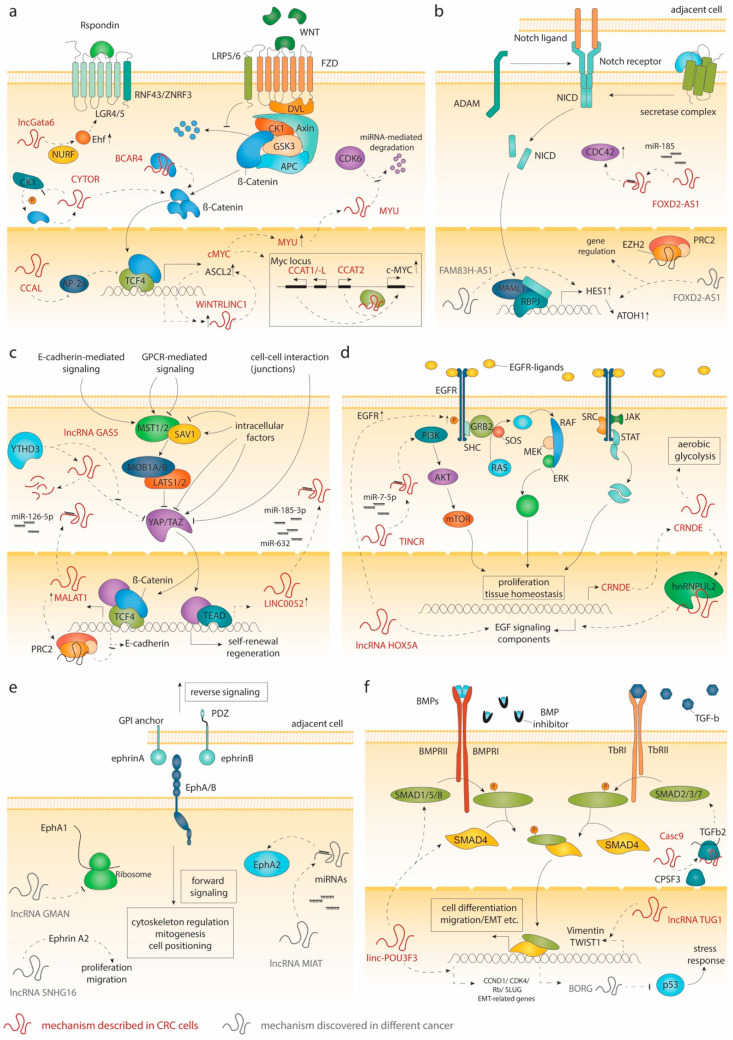
Deregulated lncRNAs involved in signaling pathways governing crypt homeostasis. Schematic representation of lncRNAs implicated in Wnt (**a**), Notch (**b**), Hippo (**c**), EGF (**d**), EphB (**e**), and BMP/TGF-β (**f**) pathways. Relative mechanisms of action are displayed.

The β-Catenin/Wnt signaling axis regulates many developmental processes as well as stem cell homeostasis in diverse tissues in adult mammals. In the intestine, this signaling cascade is essential for maintenance of ISCs and crypt homeostasis. Activation of Wnt signaling is mediated by the binding of Wnt ligands to the Frizzled (FZD)-LRP5/6 transmembrane receptor complex. Upon Wnt ligand binding, β-Catenin is released from the destruction complex comprising adenomatous polyposis coli (APC), Axin, glycogen synthase kinase 3 (GSK3), casein kinase 1 (CK1), and protein phosphatase 2A (PP2A) [3,28]. The cytoplasmic accumulation of β-Catenin leads to its translocation into the nucleus, where it acts as a co-transcription factor (TF) for T cell factor/lymphoid enhancer-binding factor (tcf/lef) (Figure 1a) [29,30,31]. This complex regulates the expression of multiple genes, including the Wnt antagonist *Axin2* [32] and *Lgr5*, a prominent marker for ISCs [33]. Tcf4 is the main downstream effector of the Wnt signaling and plays a fundamental role in the development and maintenance of ISCs, as demonstrated by knockout studies in mice [34,35]. Likewise, depletion of β-Catenin causes loss of the proliferative cells in the intestinal crypts [36]. Wnt ligands are provided by Paneth cells (small intestine) or REG4+ cells (colon) at the crypt bottom [37,38], as well as by the underlying mesenchyme [39,40]. The highest concentration is found in the vicinity of ISCs and declines along the crypt axis [41]. R-spondins and their receptors LGR4/5 [42] are pivotal for Wnt activity as depletion of *Lgr4/5* in the intestinal epithelium causes loss of ISCs [43,44]. The receptor-ligand complexes prohibit degradation of FZD receptors by RNF43 and ZNRF3 [45,46], potentiating Wnt signaling [47]. Activity of Wnt signaling is not only determined by the amount of ligand and receptor on the cell surface but also by the presence of Wnt antagonists, such as Dickkopf-1 (DKK1). Overexpression of *Dkk1* in mice induces phenotypes that are similar to *Tcf4* and *Ctnnb1* (β-Catenin) depletion models [48,49]. Altogether, these models demonstrate the need for a certain level of Wnt activity to sustain the intestinal epithelium. However, overactivation of the Wnt pathway accelerates proliferation, which is a key step in the formation of adenomas. In line with this notion, elevation of the Wnt signaling activity following inactivating APC mutations is the most frequent event in CRC development [50,51,52,53]. APC mutations disrupt the destruction complex and promote a ligand-independent activation of the Wnt transcriptional program [54,55,56]. Alternatively, CRC cases with wildtype APC usually hold mutations in other pathway components, such as β-Catenin or Axin1/2 [28].

Accumulating evidence highlights a critical role for long non-coding transcripts in the Wnt/β-Catenin cascade (Figure 1a). LncRNA lncGata6 functionally supports ISCs and its deregulation promotes CRC. The transcript is expressed in Lgr5+ stem cells and is important for stem cell maintenance; indeed, lncGata6 KO mice show reduced ISC cell counts and impaired epithelial regeneration after radiation [57]. LncGata6 recruits the NURF complex to the Ehf promoter and activates its expression. In turn, Ehf enhances Wnt activity by inducing Lgr4/5. Moreover, depletion of lncGata6 significantly reduced both adenoma formation in the APCmin mice and tumor growth in xenograft models. Treatment of patient-derived xenografts with antisense oligos further confirmed the role of lncGata6 in CRC carcinogenesis and highlights the therapeutic potential of targeting this long non-coding transcript [57]. Transcriptome profiling of patients identified several upregulated lncRNAs in CRC tissue. For example, the colorectal cancer-associated lncRNA (CCAL) promotes proliferation by suppressing AP-2alpha, a protein known to interfere with β-Catenin/tcf4 complexes in CRC cells [58]. The lncRNA BCAR4 (breast cancer anti-estrogen resistance 4), originally identified in breast cancer, has been described to directly interact with and stabilize β-Catenin protein, which enhances the expression of Wnt target genes, such as *MYC* and *CCND1*, by preventing β-Catenin degradation [59]. Interaction between β-Catenin and lncRNAs can also influence its cellular localization. Indeed, the CRC-upregulated lncRNA cytoskeleton regulator RNA (CYTOR) can promote cellular growth, epithelial to mesenchymal transition (EMT), and metastasis formation, by favoring the nuclear localization of β-Catenin. More specifically, CYTOR blocks CK1-dependent phosphorylation of β-Catenin, thereby facilitating its cytoplasmic accumulation and translocation into the nucleus. In turn, β-Catenin/tcf4 transcriptional activity regulates the expression of CYTOR, thereby forming a feed-forward regulatory loop [60].

Nuclear lncRNAs can also modulate the activity of the β-Catenin/tcf4 complex. For instance, WiNTRLINC1 (WNT-regulated lincRNA (1), an lncRNA induced by β-Catenin/tcf4, enhances the expression of *ASCL2*, a Wnt-regulated TF controlling ISC fate [31,61], by mediating the formation of a loop between its promoter and *ASCL2* regulatory region, enabling β-Catenin/tcf4 to drive *ASCL2* expression. The co-regulation of *ASCL2* and WiNTRLINC1 is increased in CRC samples and might have a role in CRC tumorigenesis [62]. Numerous ncRNA transcripts were also found to be co-regulated with the proto-oncogene *MYC*, a direct β-Catenin/tcf4 target [63]. In addition, the genomic region surrounding *MYC* contains several CRC risk loci in enhancers, which influence *MYC* expression during CRC tumorigenesis [64,65,66]. The lncRNAs CCAT1, CCAT1-L, and CCAT2 (colon cancer-associated transcript -1, -1-L, and (2), all encoded on 8q24, were identified due to their high expression levels in CRC tissue [67,68,69,70,71]. CCAT1-L is located within a super enhancer region and has been described to regulate *MYC* transcription by promoting chromatin looping between the *MYC* locus and its enhancer [71]. CCAT2 expression induces chromosomal instability, tumor growth, and metastasis formation by regulating *MYC* expression as well as other target genes. Mechanistically, CCAT2 is directly regulated by tcf4 and further potentiates its activity by physically interacting with it [68]. Because of its central role in CRC, *MYC* expression is also controlled at multiple levels. Indeed, the lncRNA GLCC1 can stabilize Myc protein levels by preventing its ubiquitination by HSP90 [72]. Interestingly, Myc itself regulates and interacts with several non-coding transcripts. For example, the lncRNA MYU (Myc upregulated lncRNA) is directly regulated by Myc. MYU positively affects CDK6-dependent cell cycle progression by protecting CDK6 mRNA from miRNA-mediated depletion [73].

Altogether, these studies prove that lncRNAs play essential roles within the Wnt/β-Catenin signaling network. The early and widespread deregulation of Wnt signaling in CRC makes functional Wnt-related lncRNAs particularly interesting as novel therapeutic targets.

### 2.2. Notch Signaling

The human Notch family comprises four receptors (Notch 1–4) and five ligands (Jagged-1, Jagged-2, and Delta-like 1, 3, and 4). When activated, receptor heterodimers undergo two proteolytic cleavages mediated by the metalloprotease ADAM10/17 and the presenilin-γ-secretase complex [74]. After its release, the Notch intracellular domain (NICD) translocates to the nucleus, where it activates RBP-Jκ-containing complexes in collaboration with the co-activator mastermind-like 1 (MAML1), leading to the regulation of genes (e.g., *HES1*) associated with EMT, proliferation, differentiation, and apoptosis (Figure 1b) [74,75].

Due to its role in the regulation of differentiation, Notch signaling is crucial for crypt homeostasis. While deletion of either Notch1 or Notch2 does not impair intestinal development, double deletion results in goblet cell hyperplasia [76,77,78]. Conversely, ectopic expression of the NICD results in repression of secretory cells and increased apoptosis of epithelial cells, with a concomitant loss of self-renewing stem cells [78,79].

Notch signaling is known to play an important role in CRC. In stem cell-like cancer cells, Notch signaling is 10- to 30-fold more active than in differentiated CRC cells. Constitutive expression of active Notch-1 promotes EMT and supports the maintenance of stem-like features [75]. Furthermore, Notch signaling has been shown to protect CRC cells from apoptosis by inhibiting p27 and ATOH1 [75]. However, Notch signaling may exert opposing functions in CRC, as suggested by the fact that while Notch 1 expression correlates with a worse overall survival, high levels of Notch2 result in a better overall survival [80]. In various cancers, oncogenic lncRNAs have been linked to the Notch signaling pathway (Figure 1b) [81,82,83]. For instance, lncRNA FOXD2-AS1 (Forkhead box D2 - antisense transcript 1) supports CRC progression by modulating the expression of genes associated with EMT and Notch signaling [84]. Although the mechanistic link between FOXD2-AS1 and Notch in CRC remains elusive, in other cancer types, FOXD2-AS1 was reported to exert oncogenic functions by interacting with EZH2, a subunit of the polycomb repressive complex 2 (PRC2) [85,86]. Alternatively, FOXD2-AS1 can sponge miR-185, which stabilizes CDC42 expression, thereby contributing to CRC cell proliferation [87]. FAM83H-AS1 (family with sequence similarity 83 member H - antisense transcript 1) is another lncRNA able to modulate the expression of Hes1, as well as Notch-1 [82]. Silencing FAM83H-AS1 can inhibit cell proliferation and migration though the exact molecular mechanisms remain undefined [88,89]. Although more efforts are needed to better understand the functions of these lncRNAs, their influence on the Notch signaling and phenotypic impacts on CRC cells suggest therapeutic potential.

### 2.3. The Hippo Pathway

The Hippo pathway, originally discovered in *Drosophila*, is key in regulating intestinal homeostasis and regeneration. Its activation can be triggered by various extrinsic (e.g., soluble factors) and intrinsic (e.g., mechanotransduction) cues, as well as via the crosstalk with other signaling pathways, including the Wnt and Notch pathways (Figure 1c). Hippo signaling is mediated by the kinases mammalian STE20-like protein kinase 1/2 (Mst1/2), Salvador homologue 1 (Sav1), the large tumor suppressor 1/2 (Lats1/2), and MOB kinase activator 1A/1B (MOB1A/1B). When activated, this kinase cascade results in the phosphorylation of Yes-associated protein (YAP), thereby promoting its interaction with the 14-3-3 protein leading to its ubiquitination and degradation. In the absence of Lats1/2 activity, YAP and its homolog transcriptional coactivator with PDZ-binding motif (TAZ) translocate to the nucleus, where they form a complex with the TEA domain family members 1–4 (TEAD1–4) to modulate the transcription of target genes controlling proliferation, migration, survival, and regeneration (Figure 1c) [90]. In the healthy intestine, the Hippo pathway was shown to be involved in stem cell self-renewal and regeneration [91,92]. In this respect, overexpression of YAP1 was found to expand the number of ISCs. In contrast, the biallelic deletion of *YAP1* did not impact normal intestinal development but severely impaired intestinal epithelium regeneration following dextran sodium sulfate (DSS) treatment [92,93]. These observations suggest that YAP1 is dispensable for normal tissue homeostasis but is essential for intestinal regeneration following injuries. In both circumstances, YAP expression levels can influence the expansion of ISCs.

Deregulation of the Hippo pathway has been implicated in the development of multiple cancers, including CRC. For instance, conditional knockout of *Mst1/2* in the intestinal epithelium of mice results in the formation of dysplasia and adenomas in the colon [93]. Similarly, conditional knockout of *Sav1* leads to the development of colonic polyps in mice. This effect is YAP dependent, as a double knockout of *Sav1* and *Yap1* did not elicit polyp formation [92]. In line with these observations, YAP was shown to be upregulated in CRC [94].

Interestingly, several lncRNAs have been implicated in the deregulation of Hippo pathway components during the initiation and progression of CRC (Figure 1c) [95]. For example, the lncRNA growth arrest-specific 5 (GAS5) interacts with and inhibits YAP activity by facilitating its degradation in healthy tissues [96]. However, in CRC cells, GAS5 expression levels are lowered due to the upregulation of YTHDF3 (YTH N6-methyladenosine RNA binding protein 3), a N6-methyladenosine (m6A) reader that facilitates the decay of methylated RNAs. The reduction of GAS5 expression levels is accompanied by a significant increase in YAP activity, which potentiates cell proliferation and invasion. Furthermore, both high levels of YTHDF3 and low levels of GAS5 correlate with a worse prognosis in CRC patients [96]. The YAP/TEAD transcriptional program also includes the regulation of lncRNAs. Among the reported target genes is the long intergenic non-coding RNA 00152 (LINC00152), which is directly regulated by YAP in CRC cells. LINC00152 competes for the binding of miRNA-185-3p and miRNA-632, which decreases the expression of F-actin and promotes cell motility and migration. Importantly, elevated levels of LINC00152 correlate with a worse prognosis for CRC patients [97]. Evidence suggests that YAP can also modulate gene expression by cooperating with the transcriptional complex β-catenin/tcf4 in intestinal epithelial cells and CRC cells [94,98]. In this context, YAP was reported to induce the expression of the lncRNA MALAT1 (metastasis-associated lung adenocarcinoma transcript 1). In turn, higher levels of MALAT1 potentiate its function as a competing endogenous RNA (ceRNA). By sponging miRNA-126-5p, MALAT1 indirectly increases levels of factors associated with cell migration, survival, and angiogenesis [94]. In addition, MALAT1 can interact with EZH2, a core subunit of PRC2, and epigenetically silence the expression of E-cadherin. These two mechanisms clearly highlight the involvement of MALAT1 in promoting molecular features associated with EMT in CRC cells. Furthermore, higher levels of MALAT1 also correlate with increased oxaliplatin resistance and a poorer overall survival [99].

Taken together, these studies highlight the important roles of lncRNAs in the regulation of the Hippo pathway and underscore their conceivable use as therapeutic targets in CRC.

### 2.4. EGF Signaling

Extracellular epidermal growth factor receptor (EGFR), also known as ErbB1/HER1, is a transmembrane tyrosine kinase that belongs to the ErbB family, and is activated by secreted ligands like EGF and TGFα. Downstream signaling pathways include the Ras/mitogen activated protein kinase (MAPK) and phosphatidylinositol 3-kinase (PI3K)/AKT/mammalian target of rapamycin (mTOR) cascade, which are involved in regulating essential cellular processes, such as proliferation and survival (Figure 1d) [100]. In the intestinal crypts, ErbB1 is highly expressed in ISCs [101]. EGF and TGFα are secreted by Paneth cells [30], as well as by the underlying mesenchyme [102]. Blocking of EGFR or MAPK in organoid models induces a reversible quiescence in Lgr5+ cells. However, these cells maintain their stem cell identity, suggesting that EGFR signaling is dispensable for the maintenance of stemness [103]. Nevertheless, balanced EGF signaling is essential for normal crypt formation as knockout mice for *Lrig1*, a negative regulator of EGFR, show severe crypt expansions and enlarged intestines [104,105]. These effects highlight the importance of tight regulation of EGF signaling for normal stem cell proliferation and tissue homeostasis.

A large number of CRCs display mutations in components of the EGF pathway, among which the most frequent are activating mutations in KRAS, BRAF, and PIK3CA [51,106]. In addition, overactivation of Wnt signaling intensifies EGF pathway activity, which is a driving force in the initiation and early progression of colon tumorigenesis. For example, ISCs harboring a KRAS mutation have an increased proliferation rate that favors the clonal expansion of KRAS mutant stem cells [107,108].

Surprisingly, little is known about lncRNAs involved in EGFR-mediated signaling in the intestinal crypt. To our knowledge, no lncRNA transcripts associated with EGFR signaling have been described in the context of normal ISCs. In CRC, expression analysis of lncRNAs associated with BRAF/KRAS mutation status revealed few transcripts with altered expression levels in tumors as compared to the normal counterpart [109]. Other studies have shown that KRAS mutation can change the composition of non-coding RNAs secreted in exosomes, potentially affecting cell–cell communication [110,111]. In addition, numerous studies have demonstrated a possible link between lncRNAs and the EGF signaling cascades in CRC cells (Figure 1d). However, these studies do not explicitly associate the function of lncRNAs to EGFR signaling or to a specific mutation in the pathway. One such example is the lncRNA TINCR (terminal differentiation-induced lncRNAs), which was initially discovered for its role in controlling human epidermal differentiation [19]. TINCR can influence tumor growth by sponging miR-7-5p, which helps to activate the PI3K/AKT/mTOR pathway [112]. The PI3K/AKT/mTOR signaling is particularly important in cancer as it controls cell proliferation, growth, motility, and metabolism [113]. Interestingly, the lncRNA CRNDE (colorectal neoplasia differentially expressed), which is expressed at higher levels in neoplastic colorectal tissue [114], has been identified as a downstream target of PI3K/AKT/mTOR and RAF/MAPK signaling in CRC. In this context, the lncRNA CRNDE induces metabolic changes in cancer cells that promote aerobic glycolysis, also known as the Warburg effect [115]. In addition, when interacting with the ribonucleoprotein hnRNPUL2, lncRNA CRNDE can enhance cell proliferation by regulating the expression of components involved in the RAS/MAPK cascade [116]. The lncRNA HOXA5 short, derived from the *HOXA6-HOXA5* locus and upregulated in advanced colon tumors, has been shown to enhance CRC growth in a xenograft mouse model. Although the mechanism remains to be clarified, HOXA5 short RNA mediates its action by modulating the expression of genes related to EGF signaling as well as by increasing the levels and phosphorylation of EGFR [117]. Up to now, the lncRNA regulatory network associated with EGF signaling has been largely unexplored. Therefore, further studies are necessary to uncover the therapeutic potential of lncRNAs in this signaling pathway.

### 2.5. EphB Signaling

Eph receptors are a large family of receptor tyrosine kinases that can interact with two classes of ligands, namely ephrin-A and -B [118]. Eph receptor-ephrin complexes form at cell–cell interfaces, where receptor–ligand engagement propagates signals in both directions (forward and reverse signaling). This signaling has been described to influence several developmental and neuronal processes, such as corticospinal tract formation, synaptic plasticity, and embryonic angiogenesis. Furthermore, it was also observed to play a role in cytoskeletal regulation, migration, mitogenesis, and cell–substrate interactions (Figure 1e) (reviewed in [119]). In the intestine, expression of EphB2/3 receptors is regulated by the β-Catenin/tcf4 complex, which generates a gradient of expression that is decreasing along the crypt–villus axis. In contrast, ephrin-B ligand levels are low at the crypt bottom and increased in the differentiated villus cells. While the gradual interaction between EphB2/ephrin-B is necessary for the positioning of precursor cells along the crypts, EphB3/ephrin-B is pivotal in the positioning of Paneth cells at the crypt bottom. Indeed, in EphB3-null mice Paneth cells are randomly distributed throughout the intestinal epithelium. Moreover, double KO of *EphB2* and -*3* has deleterious effects on the migration of precursor cells in mice, further underlining the importance for EphBs for migration and positioning of cells along the crypt–villus axis [120]. Besides its role in cell positioning, EphB2 is also involved in proliferation and cell cycle re-entry of progenitor cells [121,122]. Several studies suggest a tumor suppressive role for EphB signaling, as downregulation of EphB receptors correlates with CRC progression, tumor stages [122,123], and poor prognosis [124,125]. In this respect, a reduction of EphB receptors’ activity in APC^min/+^ mice accelerates CRC tumorigenesis, supporting a tumor suppressive role for EphB signaling [123,126]. Mechanistically, EphB activity is essential to regulate cell compartmentalization and, thus, key to restrict uncontrolled expansion of CRC cells [127].

To our knowledge, no lncRNAs have been directly linked to Eph-Ephrin signaling in the healthy intestine, nor in CRC. However, some studies have reported the interplay between lncRNAs and Eph receptors or ligands in other types of cancer (Figure 1e). For example, the lncRNA GMAN (gastric cancer metastasis-associated long non-coding RNA) partially overlaps with the ephrin-A1 gene and controls its translation by competing for the binding with GMAN-AS. Importantly, a reduction of GMAN expression in gastric cancer cell lines diminishes their invasive activity and ability to form metastases in vivo [128]. In hepatocellular carcinoma, the lncRNA MIAT (myocardial infarction-associated transcript) has been reported to regulate EphA2 levels through miRNA sponging [129]. Similarly, the lncRNA SNHG16 (small nucleolar RNA host gene) has been described as a ceRNA that can regulate ephrin-A2 levels and promote non-small cell lung cancer [130]. Interestingly, SNHG16 is expressed at higher levels in adenomas and CRC [131]. Furthermore, SNHG16 depletion reduces CRC cell proliferation and migration, as well as tumor growth in a xenograft mouse model [131,132]. However, whether the influence of SNHG16 is mediated through ephrin signaling in the context of CRC remains elusive. Taken together, the Eph-ephrin signaling is a relatively unexplored pathway with a complex two-way signal transduction that may exert oncogenic and tumor suppressive functions. Interplay between lncRNAs and Eph-ephrin signaling has yet to be discovered in the context of tissue homeostasis and CRC.

### 2.6. BMP and TGF-β Signaling

Transforming growth factor-beta (TGF-β) and bone morphogenic proteins (BMPs) are ligands that belong to the TGF-β superfamily. Binding of TGF-β and BMPs to their type II receptors TβRII and BMPRII results in phosphorylation and activation of the type I receptors TβRI and BMPRI. When activated, the type I receptor can phosphorylate and activate different downstream Smad proteins. While initially divergent, these pathways eventually converge by forming complexes with Smad4, which subsequently translocate to the nucleus to regulate gene expression (Figure 1f) [133]. Under normal conditions, TGF-β has many roles in wound healing, immune regulation, and restriction of epithelial cell proliferation in the intestine [134,135]. Due to its vast array of functions, crosstalk between TGF-β and other pathways, including the BMP, Hippo, and Notch pathways, is paramount to its physiological effect [136]. In addition, the effect of TGF-β signaling differs based on the specific cell type, as regulation by Smads can depend on the presence of different DNA-binding partners. Expression of NANOG or Oct4 elicits a stem-like phenotype, while cells expressing MYOD1 promote a more differentiated muscle-cell phenotype [136,137]. Relatively little is known about the implications of TGF-β signaling in the context of the intestinal stem cell niche. However, loss of TGF-βRII appears to impair crypt fission, crypt regeneration, and differentiation of ISCs towards Paneth cells [138]. BMPs help to maintain intestinal tissue homeostasis by restricting ISCs hyperproliferation and by promoting epithelial cell differentiation [138,139]. Secreted by mesenchymal cells located beneath the epithelium, they mediate their function by gradually opposing WNT activity along the crypt–villus axis.

In CRC, TGF-β has long been known to increase metastasis formation [140]. Furthermore, sequential increases in SMAD2 and H-Ras levels seem to be driving factors of metastasis in CRC [141]. In contrast to these indications, loss of TGF-β2 can result in an increase of colonic adenomas and carcinomas [142]. This ambivalent role of TGF-β signaling likely depends on CRC stage and is influenced by critical oncogenic events. For instance, mutation of p53 favors TGF-β pro-tumorigenic effects [143]. Loss of Smad4 was also shown to elicit similar changes [144].

Importantly, in CRC, TGF-β and BMP signaling pathways can be influenced by deregulated lncRNAs (Figure 1f). For instance, the lncRNA cancer susceptibility 9 (CASC9), upregulated in 70% of primary CRC tumors, promotes both CRC tumor growth and resistance to apoptosis [145]. CASC9 interacts with the endonuclease CPSF3 and modulates the stability of various mRNAs, including TGFβ2, which ultimately impacts the phosphorylation of Smad3 [145]. CASC9 was also implicated in the progression and chemoresistance of many other cancers, including liver cancer [146], esophageal cancer [147], and gastric cancer [148]. Similarly, the TGF-β-sensitive lncRNA taurine upregulated-1 (TUG1), which is upregulated in CRC tissue, is crucial in promoting cell migration [149]. TUG1 assists TGF-β-mediated EMT and metastasis formation by regulating the expression of vimentin and TWIST1. Moreover, TUG1 knockdown can reduce the pro-migratory function of TGF-β [149]. The BMP/OP-responsive gene (BORG) is another example of lncRNAs regulated by TGF-β/BMP signaling pathways. First discovered in the context of breast cancer [150], BORG was also detected in the plasma of CRC patients receiving carboplatin therapy [151]. Upregulation of BORG in response to carboplatin treatment induces chemoresistance [151]. However, while TGF-β/BMP-dependent regulation of BORG was demonstrated in breast cancer, the link between BORG upregulation in carboplatin-treated CRC patients and these pathways remains unclear.

Additionally, the BMP transcriptional program utilizes lncRNAs to support tumor progression. For example, the lncRNA linc-POU3F3 (also known as PANTR1 - POU3F3 adjacent non-coding transcript 1) was shown to support CRC cell survival, proliferation, and dissemination by influencing the expression of several genes, including caspase-3/9, cyclin D1, cyclin-dependent kinase 4 (CDK4), retinoblastoma (Rb), and SLUG [152]. Interestingly, while linc-POU3F3 inhibition decreases the CRC cell migratory potential by lowering the expression of EMT-related genes (e.g., N-cadherin, vimentin, SLUG, and SNAI1), it concomitantly activates the BMP signaling pathway. In this context, it was proposed that linc-POU3F3 is important to prevent the BMP pathway from activating cell autophagy [152]. Finally, in a recent whole transcriptome sequencing study comparing normal tissue with early stage (ES) and late-stage (LS) CRCs, the lncRNA MSTRG.35002 was predicted to be a key lncRNA in the progression of CRC [153]. Moreover, functional enrichment analysis highlighted a close association between the lncRNA MSTRG.35002 and the BMP signaling signature [153]. While this type of study is largely speculative, it nevertheless represents an interesting method that can facilitate the functional assignment of unknown lncRNAs.

## 3. LncRNA-Directed Therapeutic Approaches

Inhibition of signaling pathways that are essential for CRC cells is key to counteract tumor growth and progression. For instance, therapeutic agents that can block Wnt signaling are of great interest, as hyperactivation of Wnt is a recurrent feature observed in a large proportion of CRCs. So far, most Wnt signaling inhibitors are directed against components acting upstream of APC (e.g., frizzled receptors, R-spondin 3, and porcupine), and are therefore unlikely to be effective in CRC, where APC is frequently mutated [154]. On the other hand, molecules interfering with β-catenin (e.g., ICG-001 and PRI-724), which could have therapeutic potential in CRC, are likely to disrupt normal stem cell function, thus compromising tissue homeostasis. Interestingly, lncRNAs are potent regulators of a wide range of cellular processes with high cell or tissue/tumor specificity. In this respect, lncRNA-targeted therapy could advantageously impair specific signaling cascades in cancer cells while avoiding or minimizing adverse effects on healthy cells. Recent advances in oligonucleotide-based therapy and CRISPR technology bring forward numerous tools and strategies, which could be pivotal in the development of novel therapeutic approaches that aim to interfere with oncogenic lncRNAs (Table 1).

### 3.1. RNAi

**Figure 2:**
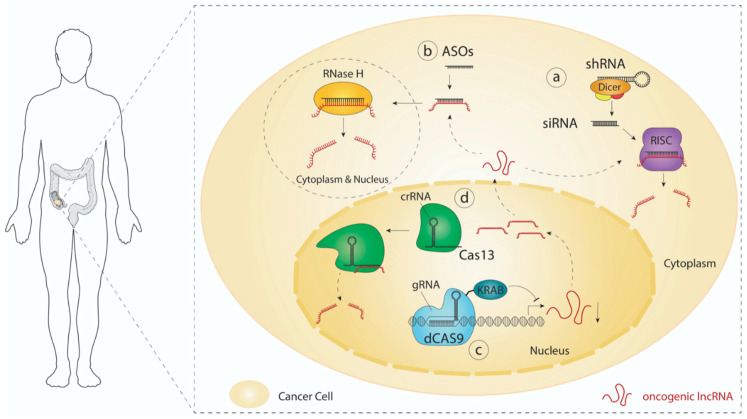
Strategies to interfere with oncogenic lncRNAs. (**a**) RNA interference. Short hairpin RNAs (shRNAs) are first processed by DICER, which removes the terminal loop to generate small interfering RNAs (siRNAs). These are further incorporated into the RNA-induced silencing (RISC) complex to mediate the degradation of complementary sequences. (**b**) Antisense oligonucleotides. ASOs catalyze the cleavage of their complementary target RNAs via the ribonuclease H in both the cytoplasm and the nucleus. (**c**) CRISPR interference. The dCas9-KRAB/gRNA complex mediates the epigenetic silencing of targeted genes. (**d**) CRISPR Cas13. Guided by a CRISPR-RNA (crRNA), the ribonuclease Cas13 catalyzes the cleavage of single stranded RNA.

Modulation of gene expression using RNA interference or RNAi was first discovered in *C. elegans* by Andrew Fire and Craig Mello [155]. This method relies on the intracellular delivery of short exogenous double-stranded RNA molecules (dsRNA). When supplied as short hairpin RNAs (shRNAs), molecules are first recognized and processed by the RNAse III enzyme DICER, which removes the terminal loop to generate 21–25-nucleotide dsRNA molecules or small interfering RNAs (siRNAs). One strand of the RNA duplex is then incorporated into the multiprotein complex known as the RNA-induced silencing complex (RISC) [156]. Once loaded with siRNA, the RISC complex can hybridize with and degrade complementary target RNAs by using its endonuclease activity (Figure 2a). Since its discovery, dsRNA-mediated interference has been extensively used to better understand the function of genes and their roles in various biological processes in vitro [157,158], and multiple studies have reported efficient delivery of siRNAs to target protein-coding transcripts in vivo [159,160]. Evidence also suggests that siRNAs are able to silence lncRNAs in vivo [161,162]. However, many challenges, such as their stability, specificity, immunogenicity, toxicity, and delivery, still prevent the widespread clinical use of RNAi-based therapy. Remarkably, most of these hurdles can be resolved by introducing various chemical modifications at the ribose (e.g., 2′-O methyl), phosphate (e.g., phosphorothioate), and base (e.g., pseudouridine) level. Fine-tuning the type, the position, and the proportion of modified nucleotides can increase their stability and specificity, while reducing their immunogenicity and toxicity [163,164]. Despite many challenges, Alnylam^®^ Pharmaceuticals (Cambridge, MA, USA) recently obtained the FDA’s approval for the first RNAi-based therapy. Patisiran (Onpattro^®^), formulated as lipid nanoparticles (LNPs) containing short interfering RNA, can now be used for the treatment of hereditary transthyretin amyloidosis in adults [165]. Importantly, this novel strategy is particularly interesting as it provides a blueprint that could be adapted for the design of lncRNA-targeted therapies.

### 3.2. Antisense Oligonucleotides (ASOs)

ASOs are synthetic single-stranded DNA or RNA oligos (15 to 25 nucleotides in length) that can bind and catalyze the cleavage of complementary target RNAs. Differently from siRNAs, which are loaded into Ago2 to activate the RISC, the DNA:RNA hybrid formed between ASOs and their cognate sites are substrates for the ribonuclease H (RNAse H). Importantly, RNAse H localizes to both the cytoplasm and the nucleus, thus allowing RNA targeting in both compartments (Figure 2b) [166]. To improve cellular internalization and reduce sensitivity to nucleases, ASOs also require chemical modifications. As for siRNAs, these changes can target phosphates, riboses, and bases. However, beside the phosphorothioate (PS), locked nucleic acid (LNA), and 5′methylcytosine (5mc) modifications, most chemical changes inhibit the recruitment of and activity of RNAse H [167,168]. While modifications like 2′-O methyl (2′O-Me), and phosphorodiamidate morpholino oligomers (PMOs) would most likely block the activity of RNAse H by steric hindrance, they could be exploited to promote conformational/structural changes or to interfere with specific interactions mediated by an RNA of interest. Modified ASOs, such as PS-ASOs, have been shown to easily interact with abundant plasma proteins, which facilitates tissue distribution and reduces renal clearance [169,170,171]. However, ASOs with high propensity to bind multiple proteins are generally associated with more toxicity [172,173].

Several reports have demonstrated the potential of ASOs against lncRNAs in various mouse models. For example, silencing the lncGATA6 with intratumoral injection of ASOs in CRC patient-derived xenograft (PDX) models has been shown to significantly reduce tumor growth [57]. Subcutaneous injection of ASOs targeting the lncRNA MALAT1 is sufficient to reduce the formation of breast and lung cancer metastasis in MMTV-PyMT mouse mammary carcinoma and lung cancer xenograft models, respectively [174,175]. In an Angelman syndrome mouse model, intracerebroventricular (ICV) injection of ASOs, directed against the long non-coding RNA UBE3A antisense transcript (UBE3A-ATS), could help to restore UBE3A protein levels, which contributes to alleviate part of the cognitive deficits associated with the disease [176]. Up to date, multiple ASO-based drugs have already been approved by the FDA [177]. Among these RNA therapeutics, Eteplirsen and Golodirsen influence the splicing of dystrophin to treat Duchenne muscular dystrophy (DMD), while Nusinersen promotes a full-length splice variant of SMN2 mRNA that is effective in reducing the symptoms associated with spinal muscular atrophy (SMA). Importantly, most ASO therapeutics have been approved in the last 5 years, which highlight the growing potential of this technology.

### 3.3. CRISPR Technology

Emmanuelle Charpentier and Jennifer Doudna were awarded the 2020 Nobel prize in Chemistry for the discovery of CRISPR/cas9 gene editing system. Since their breakthrough publication in 2012 [178], the CRISPR/cas9 technology has quickly emerged as a powerful tool to edit the genome or influence gene expression. One particularly interesting development is the CRISPR interference (CRISPRi) system, which could be used to silence the transcription of oncogenic lncRNAs. In this system, a catalytically dead Cas9 (dCas9) is fused to a Krüppel-associated box (KRAB) repression domain, which is used to epigenetically repress the transcription of specific genomic loci (Figure 2c). Genome-wide CRISPRi screens targeting either human protein-coding genes or lncRNAs showed the simplicity and the efficacy of this technology to control gene expression [179,180]. Recently, the RNA-targeting CRISPR-Cas13 system emerged as another exciting tool to specifically disrupt oncogenic transcripts (Figure 2d) [181]. Evidence also suggests that the Cas13-based system is efficient in targeting lncRNAs [182].

However, CRISPRi- or Cas13-based therapeutic approaches face several challenges. One of the major limitations is the efficient delivery of a large cargo size (dCas9-KRAB or Cas13 and a guide RNA) to the cells of interest, using viral or nonviral vectors [183]. Among viral delivery systems, adeno-associated viruses (AAVs) exhibit interesting features, such as high gene transfer efficiency and low immunogenicity. However, AAVs have small packaging capacity (<5 kb), which limits their attractiveness for CRISPR-based approaches [183,184]. Alternatively, nonviral delivery systems, including lipid- (LNPs) and polymer-based nanoparticles (PNPs), have substantially evolved in the past decades [185]. Recent advances demonstrate that efficient and even tissue-specific delivery of Cas9 mRNA and guide RNAs can be achieved using engineered LNPs [186,187]. Up to now, multiple ongoing clinical trials are using CRISPR technology to treat various diseases and disorders, including cancer [188]. While most cancer-associated studies aim at generating ex vivo modified T cells for the treatment of various cancer types, novel CRISPR-based approaches, including CRISPR-directed lncRNA therapies, will certainly surface in the coming years.

## 4. Conclusions

LncRNAs are increasingly recognized as critical regulators of numerous cell functions. In the intestine, lncRNAs modulate several signaling pathways, which are pivotal in maintaining tissue homeostasis. In contrast, their deregulation in diseases, such as cancer, can rewire these signaling cascades to enable malignant cells to proliferate and disseminate. Because of their high tissue (tumor) specificity, lncRNA-targeted therapy represents an exciting avenue to specifically disrupt important signaling pathways, such as the Wnt, Notch, and Hippo pathways, in tumor cells without harming their essential functions in normal tissues. Molecular tools, such as siRNAs, ASOs, and CRISPR technology, are now opening the race for the development of RNA-based therapeutics that target oncogenic lncRNAs.

## Figures and Tables

**Table 1:** CRC-associated oncogenic lncRNAs involved in key signaling pathways.

LncRNA	Phenotype	Signaling Pathway	Mechanism	References
lncGata6	Stem cells, Tumorigenesis	Wnt	Enhances WNT activity	[57]
CCAL	Proliferation, Invasion, Migration	Wnt	Stabilizes β-Catenin/TCF-4 complex by suppression of AP-2alpha	[58]
BCAR4	Proliferation, Migration	Wnt	Stabilization of β-Catenin	[59]
CYTOR	Proliferation, EMT	Wnt	Stabilization of β-Catenin	[60]
MYC locus (CCAT1, CCAT1-L, CCAT2)	Proliferation, Metastasis, Chromosomal instability	Wnt	Regulation of c-MYC expression	[64,65,66,67,68,69,70,71]
GLCC1	Proliferation, Survival, Glycolysis	Wnt	Stabilization of c-Myc protein	[72]
MYU	Cell cycle, Proliferation	Wnt	Interacts with hnRNP-K and stabilizes CDK6 mRNA	[73]
FOXD2-AS1	Proliferation, Invasion, Migration	Notch	Regulation of EMT and Notch signaling	[84]
FAM83H-AS1	Proliferation, Migration	Notch	Modulates Notch signaling	[88,89]
LINC00152	Cell motility, Migration	Hippo	miRNA sponging	[97]
MALAT1	Survival, Migration, EMT	Hippo	miRNA sponging; Epigenetic silencing of targets	[94,99]
TINCR	Proliferation, Metastasis formation	EGF	miRNA sponging	[112]
CRNDE	Warburg effect, Proliferation	EGF	Activates RAS\MAPK pathway; Interacts with hnRNPUL2	[115,116]
HOXA5	Proliferation	EGF	Modulates EGF signaling	[117]
CASC9	Anti-apoptotic, Proliferation	BMP/TGF-β	Interacts with endonuclease CPSF3	[145]
TUG1	EMT, Metastasis formation	BMP/TGF-β	Regulates expression of Vimentin and TWIST1	[149]
linc-POU3F3	Survival, Proliferation, Migration	BMP/TGF-β	Regulates EMT-associated genes, Inhibits BMP signaling	[152]
